# Retraction: Physical activity and glioblastoma: a paradigm shift in neuro-oncology therapy

**DOI:** 10.3389/fonc.2026.1869444

**Published:** 2026-05-07

**Authors:** Frontiers Editorial Office

The Journal retracts the 30 July 2025 article cited above.

Following publication, concerns were raised about the citations and scientific validity of the article. An investigation, conducted in accordance with Frontiers’ policies, found evidence of undisclosed use of generative AI in the preparation of the manuscript, including conversational artefacts characteristic of large language model output that were not removed prior to publication. As a result, the conclusions of the article have been deemed unreliable and the article has been retracted.

This retraction was approved by the Chief Editors of Frontiers in Oncology and the Chief Executive Editor of Frontiers. The authors received a communication regarding the retraction and had a chance to respond. This communication has been recorded by the publisher.

